# Selective Denervation of the Facial Dermato-Muscular Complex in the Rat: Experimental Model and Anatomical Basis

**DOI:** 10.3389/fnana.2021.650761

**Published:** 2021-03-22

**Authors:** Vlad Tereshenko, Dominik C. Dotzauer, Udo Maierhofer, Christopher Festin, Matthias Luft, Gregor Laengle, Olga Politikou, Holger J. Klein, Roland Blumer, Oskar C. Aszmann, Konstantin D. Bergmeister

**Affiliations:** ^1^Clinical Laboratory for Bionic Extremity Reconstruction, Department of Plastic, Reconstructive and Aesthetic Surgery, Medical University of Vienna, Vienna, Austria; ^2^Center for Biomedical Research, Medical University of Vienna, Vienna, Austria; ^3^Department of Plastic Surgery and Hand Surgery, University Hospital Zurich, Zurich, Switzerland; ^4^Center for Anatomy and Cell Biology, Medical University of Vienna, Vienna, Austria; ^5^Department of Plastic, Reconstructive and Aesthetic Surgery, Medical University of Vienna, Vienna, Austria; ^6^Department of Plastic, Aesthetic and Reconstructive Surgery, University Hospital St. Poelten, Karl Landsteiner University of Health Sciences, Krems, Austria; ^7^Department of Plastic, Aesthetic and Reconstructive Surgery, University Hospital St. Poelten, Krems, Austria

**Keywords:** facial nerve, trigeminal nerve, motor-sensory control, facial muscles, axon quantification, whole-mount staining

## Abstract

The facial dermato-muscular system consists of highly specialized muscles tightly adhering to the overlaying skin and thus form a complex morphological conglomerate. This is the anatomical and functional basis for versatile facial expressions, which are essential for human social interaction. The neural innervation of the facial skin and muscles occurs via branches of the trigeminal and facial nerves. These are also the most commonly pathologically affected cranial nerves, often requiring surgical treatment. Hence, experimental models for researching these nerves and their pathologies are highly relevant to study pathophysiology and nerve regeneration. Experimental models for the distinctive investigation of the complex afferent and efferent interplay within facial structures are scarce. In this study, we established a robust surgical model for distinctive exploration of facial structures after complete elimination of afferent or efferent innervation in the rat. Animals were allocated into two groups according to the surgical procedure. In the first group, the facial nerve and in the second all distal cutaneous branches of the trigeminal nerve were transected unilaterally. All animals survived and no higher burden was caused by the procedures. Whisker pad movements were documented with video recordings 4 weeks after surgery and showed successful denervation. Whole-mount immunofluorescent staining of facial muscles was performed to visualize the innervation pattern of the neuromuscular junctions. Comprehensive quantitative analysis revealed large differences in afferent axon counts in the cutaneous branches of the trigeminal nerve. Axon number was the highest in the infraorbital nerve (28,625 ± 2,519), followed by the supraorbital nerve (2,131 ± 413), the mental nerve (3,062 ± 341), and the cutaneous branch of the mylohyoid nerve (343 ± 78). Overall, this surgical model is robust and reliable for distinctive surgical deafferentation or deefferentation of the face. It may be used for investigating cortical plasticity, the neurobiological mechanisms behind various clinically relevant conditions like facial paralysis or trigeminal neuralgia as well as local anesthesia in the face and oral cavity.

## Introduction

Facial muscles play a pivotal role in mastication, respiration, articulation, corneal cell protection, and thus survival. Moreover, the vast variety of facial expressions are essential for human social interaction, which is an indispensable part of human culture (Schmidt and Cohn, 2001). This functionality requires a complex facial dermato-muscular system, which is based on a three-dimensional architecture of facial muscles interacting with each other and the overlaying skin (Happak et al., 1997; Sandulescu et al., 2019). To power such a complex and highly functional interplay, the facial muscles' differ from other skeletal muscles regarding their function, motor control and sensory feedback (Van Buskirk, 1945; Cattaneo and Pavesi, 2014).

In detail, facial muscles are innervated by the extracranial facial nerve, which is considered a pure motor nerve (Bowden and Mahran, 1960; Cattaneo and Pavesi, 2014). This pure efferent innervation implies a lack of conventional proprioceptive feedback from the facial musculature via the facial nerve, which is not seen in other skeletal muscles (Arends and Dubbeldam, 1982; Happak et al., 1994; Proske and Gandevia, 2012; Cattaneo and Pavesi, 2014). This notion was supported by evidence demonstrating the lack of muscle spindles in facial muscles of different Mammalia as well as humans (Stål et al., 1987, 1990). Some contradictory anatomical and electrophysiological data indicates that sensory feedback from facial muscles may be transduced via mechanoreceptors of the facial skin (Trulsson and Johansson, 2002; Siemionow et al., 2011; Proske and Gandevia, 2012; Frayne et al., 2016). Nevertheless, no conclusive data exists regarding the nature of sensory feedback from facial muscles (Cattaneo and Pavesi, 2014).

Therefore, the trigeminal and facial nerves in the rat are highly relevant structures in neurological research. Various experimental models for researching the facial nerve (Kreutzberg et al., 1989; Moran and Graeber, 2004, Guntinas-Lichius et al., 2011; Olmstead et al., 2015) the trigeminal nerve (Titmus and Faber, 1990; Pavlov et al., 2008; Bendella et al., 2011; Bregman et al., 2014; Ding et al., 2017; Dingle et al., 2019) have been established. Despite this widespread research surrounding cranial nerves, currently, there is no comprehensive surgical model for total deafferentation or deefferentation of superficial facial structures established in the rat.

In this study, we demonstrate a universal experimental model in the rat to investigate the selective impact of the afferent and efferent components on the complex facial system. Specifically, we provide two reproducible surgical models to completely deprive the facial skin or musculature of either efferent or afferent innervation. Furthermore, we performed a detailed description for harvesting three facial muscles (levator labii superioris, dilator nasi, and levator auris longus muscles) for neuromuscular research purposes, with subsequent immunofluorescent whole-mount staining. Lastly, we quantified the axonal components in all cutaneous branches of the trigeminal nerve.

## Materials and Methods

### Experimental Design

**Figure 1:**
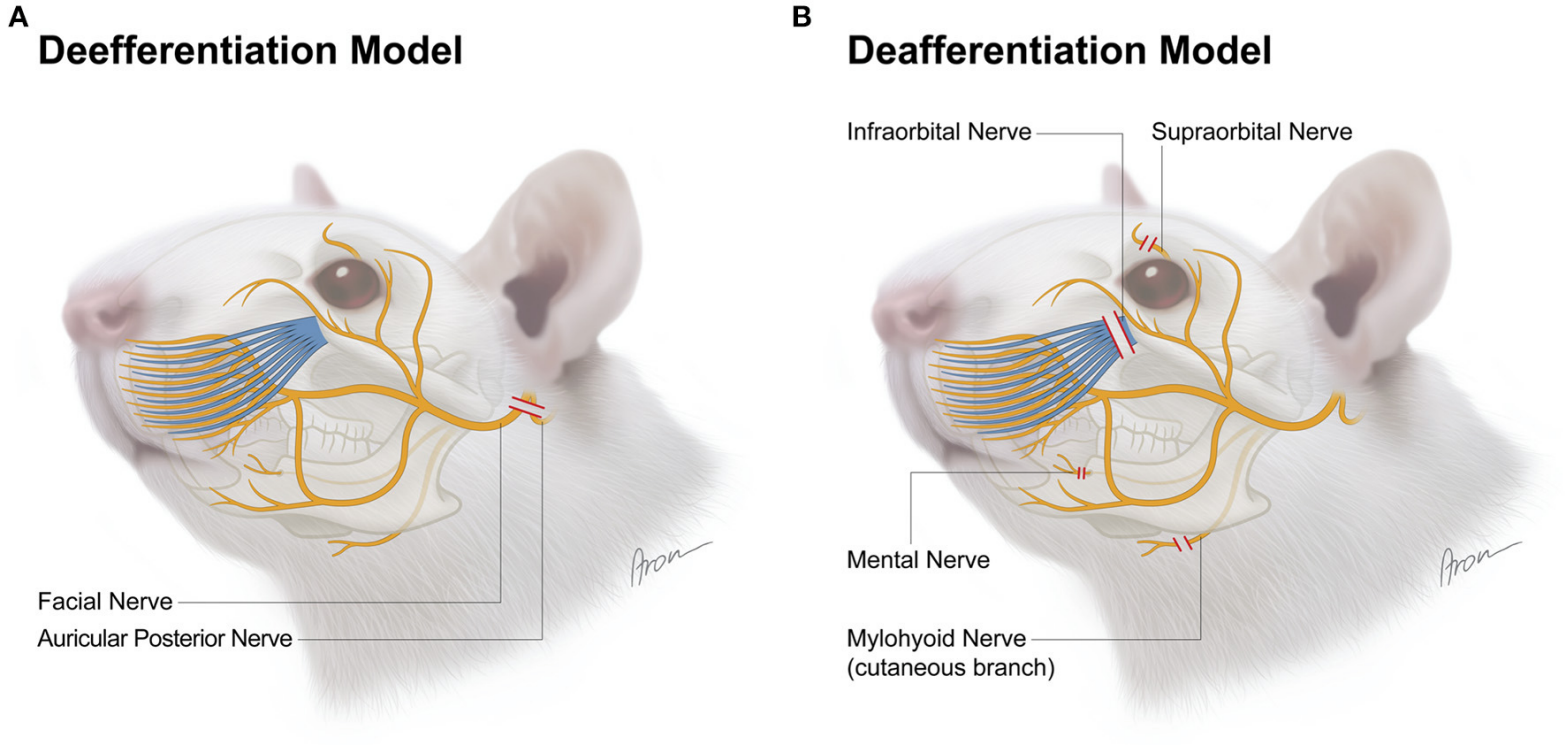
Surgical model for deefferentation **(A)** and deafferentation **(B)** of facial structures in the rat. The red lines indicate transection level for each nerve.

Eighteen male Sprague-Dawley rats aged 8 to 10 weeks were used in this study. Rats were split into one pilot group (*n* = 4) to establish the anatomical dissection and two main groups: in group A (*n* = 7) deafferentation and in group B (*n* = 7) deefferentation was performed (Figure 1). During the denervation procedure, the afferent nerves were harvested for immunofluorescent staining. Subsequently, whisker pad movement in both groups was recorded 4 weeks after surgery. In both groups, ipsilateral facial muscles (levator labii superioris, dilator nasi, and levator auris longus muscles) were harvested 4 weeks after surgery for immunofluorescent staining. Approval was obtained from the ethics committee of the Medical University of Vienna and the Austrian Ministry for Research and Science (reference number BMWF- 66.009/0302-V/II/3b/2019).

### Surgical Model

All procedures were performed under standardized conditions. Preoperatively, ketamine (100 mg/kg) and xylazine (5 mg/kg) were injected i.p. Afterwards, anesthesia was induced by inhalation of isoflurane via an endotracheal tube and subcutaneous piritramide injections (0.3 mg/kg) were administered for analgesia which allowed for uneventful surgery. For postoperative analgesia, piritramide and glucose were added to the drinking water (two ampules Dipidolor equaling 30 mg piritramide + 10 mL 10% glucose solution in 250 mL drinking water) for 5 days. All procedures were performed by the same surgeon and one assistant.

#### Deefferentation Model

**Figure 2:**
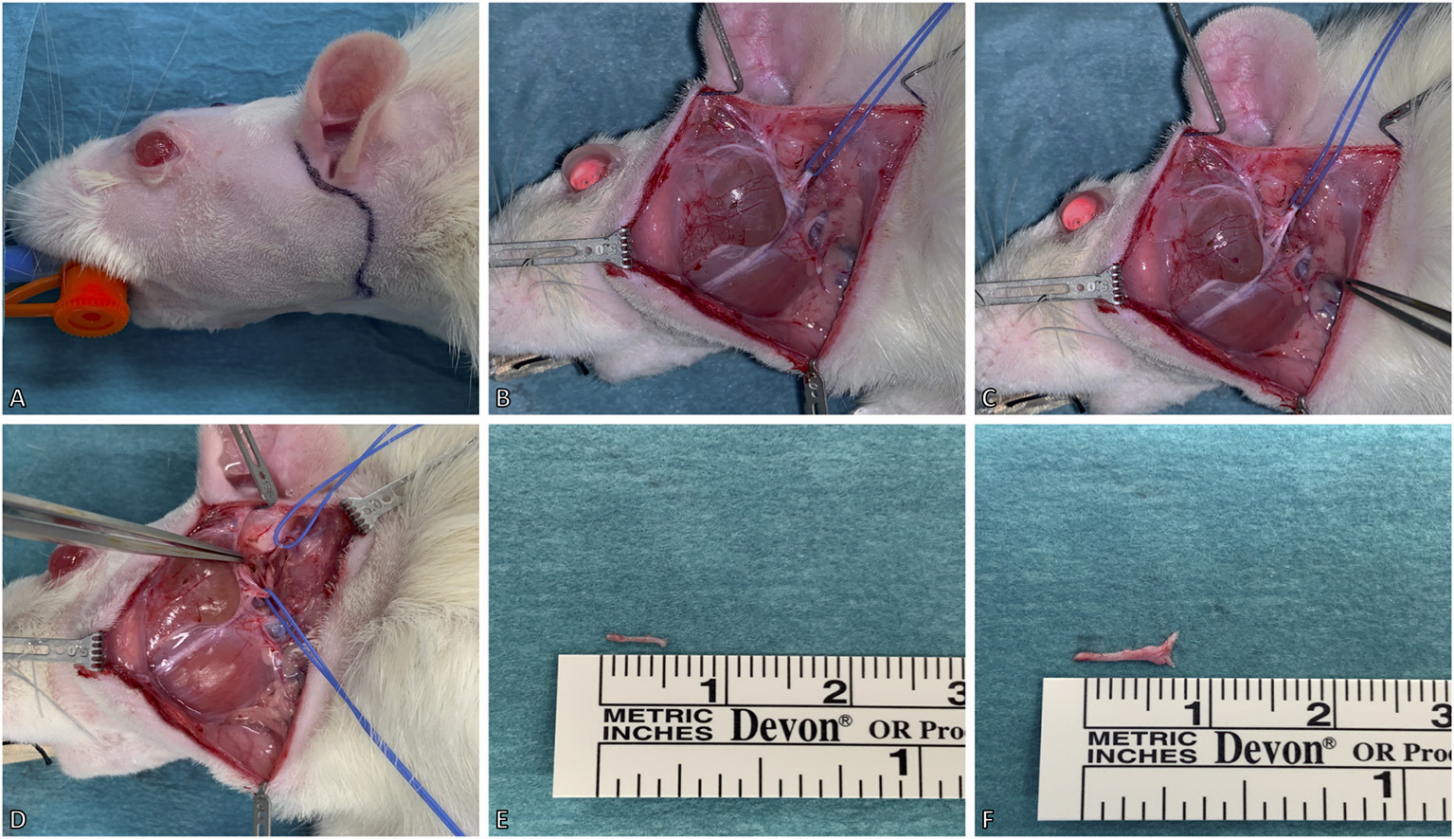
Surgical model for deefferentation of the facial nerve. **(A)** A preauricular incision was made up to the ventral border of the masseteric muscle. **(B)** The facial nerve (slung with the blue vessel loop) was isolated between the extraorbital lacrimal and parotid glands. The marginal mandibular and buccal branches of the facial nerve were identified superficial to the masseteric muscle. **(C)** For better visualization of the facial nerve, the temporal superficial vein was cauterized. **(D)** Lastly, the facial nerve's main trunk was dissected and isolated to its exit from the stylomastoid foramen, where the posterior auricular nerve (slung with the upper vessel loop) was identified. **(E,F)** The excised main trunk of the facial nerve measured 8 mm and the posterior auricular nerve 5 mm in length. The animal's eyes were protected with an ointment.

In this model, the facial nerve was cut unilaterally for deefferentation of the facial muscles. A pre-auricular incision was made at the ventral border of the masseteric muscle (Figure 2A). The facial nerve was then approached via blunt dissection between the extraorbital lacrimal and rostral border of the parotid glands. Further dissection was performed using a Zeiss operating microscope (Munich, Germany). The auricular cartilage was isolated, followed by careful dissection beneath the extraorbital lacrimal gland to visualize two distal branches (buccal and marginal mandibular) of the facial nerve which run superficial to the masseteric muscle. The facial nerve was identified by proximal dissection of these branches beneath the posterior facial vein (Figure 2B). For better visualization of the facial nerve, the anterior facial vein was cauterized (Figure 2C). The main trunk was then further dissected to its exit from the stylomastoid foramen which was followed by the identification of the posterior auricular nerve. During this process, particular caution is essential to prevent damage to the superficial temporal artery underneath the facial nerve. Vessel loops were used for atraumatic nerve manipulation during dissection (Figure 2D). Afterwards, both nerves were transected as close as possible to the stylomastoid foramen and each distal stump was then further resected by ~6–9 mm (Figures 2E,F). Furthermore, the stylomastoid foramen as well as the distal stumps of all branches of the facial nerve were cauterized to prevent any spontaneous regeneration.

#### Deafferentation Model

**Figure 3:**
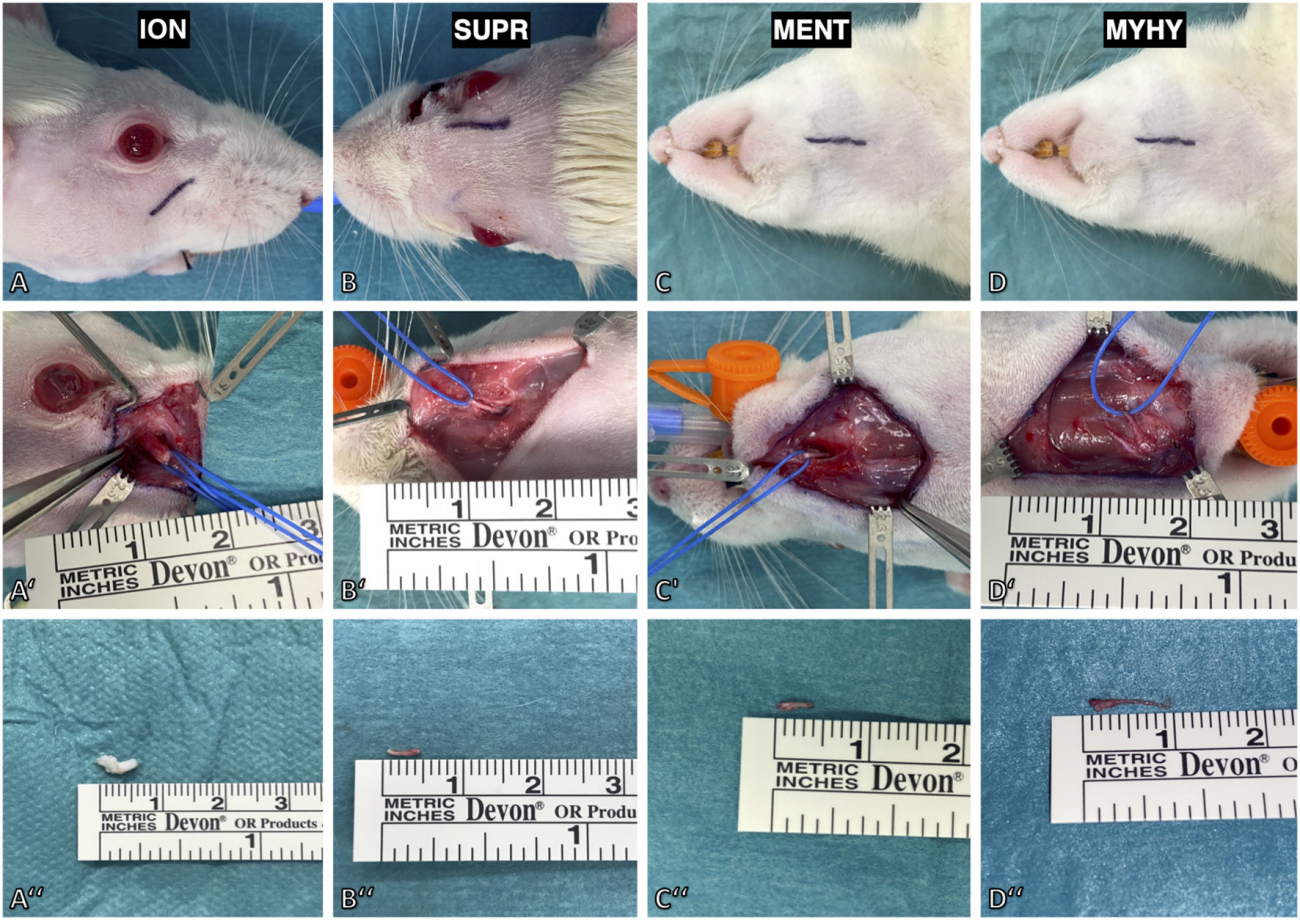
Surgical model for deafferentation of the face in rat. **(A)** The infraorbital nerve (ION) was exposed via an incision below the infraorbital ridge. **(A')** Maximal length of the infraorbital nerve was achieved by slinging a vessel loop around it followed by dissection to its exit from the infraorbital foramen. **(A”)** The excised infraorbital nerve measured 5 mm. **(B)** The supraorbital nerve (SUPR) was exposed via a paramedian incision 2 mm median to the upper eyelid. **(B',B”)** After dissecting it to the supraorbital foramen, the nerve was isolated and excised with a length of 5 mm. **(C)** The mental nerve (MENT) and cutaneous branch of the mylohyoid nerve were approached by a submandibular incision. **(C')** The mental nerve was isolated by lifting the masseteric muscle from the premaxilla. **(D')** The cutaneous branch of the mylohyoid nerve (MYHY) was identified at its median exit in-between the digastric muscles and is transected at its ramification point in the submental skin area. **(C”,D”)** The excised mental nerve measured 4 mm and the cutaneous branch of the mylohyoid nerve 9 mm.

In this model, four afferent nerves of the trigeminal nerve innervating the majority of the facial skin of the rat were exposed and subsequently unilaterally transected. First, the infraorbital nerve was exposed outside the orbital cavity via a 1 cm incision parallel to the zygomatic arch ~4 mm below the eyeball (Figure 3A). The nerve was identified through blunt dissection along the superficial fascia of the masseter muscle toward the infraorbital foramen. It was then isolated further distally toward the whisker pad using vessel loops (Figure 3A'). Following dissection, the infraorbital nerve was transected at its exit from the infraorbital foramen and then resected by ~6 mm (Figure 3A”). Afterwards, the supraorbital nerve was exposed via a 1 cm paramedian incision 2 mm median to the upper eyelid (Figure 3B). The supraorbital nerve is dissected to its branching point and resected by ~5 mm (Figures 3B',B”). To access the mental and mylohyoid nerves, a submandibular incision was made along the lateral border of the mandible (Figure 3C). The mental nerve was identified by bluntly separating the masseter muscle from the right anterior mandible to the masseteric ridge (Figure 3C'). This was followed by dissection of the nerve toward its distal branching point and resection by ~6–7 mm (Figure 3C”). Lastly, the cutaneous sensory branch of the mylohyoid nerve was identified due to it emerging 1 mm lateral to the median line separating the two digastric muscles (Figure 3D). Similar to the aforementioned afferent nerves, it was also resected by ~9 mm (Figures 3D',D”). Afterwards, the cranial foramina of the emerging afferent nerves as well as their distal stumps were cauterized to prevent spontaneous regeneration. Wound closure was done via deep dermal sutures with 6–0 Vicryl (Ethicon, Inc.; New Jersey, USA) followed by a running skin suture with 4–0 Vicryl (Ethicon, Inc., New Jersey, USA). Operation time and any complications were documented for each procedure.

### Axon Quantification of the Afferent Nerves

Axon quantification of afferent nerves innervating the facial skin was performed using an immunofluorescent staining protocol as previously described (Gesslbauer et al., 2017). Briefly, after harvesting, nerve samples were fixated by immersion in 4% paraformaldehyde (PFA) for no longer than 20 h and then stored in 0.1 M phosphate buffered saline (PBS) for 24 h at +4°. Subsequently, the samples were dehydrated in a series of sucrose/PBS solutions with increasing sucrose concentrations (10, 25, and 40%) and embedded in Tissue-Tek® O.C.T.™ Compound (Sakura Finetek Europe B.V., Alphen aan den Rijn, Netherlands). The nerve samples are cut into 10 μm thick sections using a Cryotom (Leica CM3050 Cryostat, Leica Biosystems, Germany) mounted on Superfrost Ultra Plus® microscope slides (Thermo Scientific/Menzel). Fluorescence staining was performed using primary antibodies against choline acetyltransferase (anti-ChAT, Merck Millipore/Chemicon International, Temecula, CA; catalog number AB144P) and neurofilament (anti-NF, Merck Millipore/Chemicon International; catalog number AB5539). Images of nerve cross sections were acquired using a fully integrated imaging system (TissueFAXS; TissueGnostics, Vienna, Austria). Automated quantification of axons within the afferent nerves was performed using StrataQuest version 5.1.249 and TissueQuest version 4.0.1.0128 (TissueGnostics, Vienna, Austria). Lastly, images were acquired using an inverted point laser scanning confocal microscope (LSM780, Carl Zeiss, Germany). Descriptive statistics were calculated for all nerve specimens. Data is presented as mean ± standard deviation.

### Functional Assessment and Video Recording

A qualitative functional assessment of whisker pad movement was performed via video recordings 4 and 12 weeks after surgery as established by several previous publications (Carvell and Simons, 1990; Guntinas-Lichius et al., 2002; Tomov et al., 2002; Angelov et al., 2007; Kiryakova et al., 2010; Soehnchen et al., 2010). The rats were placed into a stress-free environment for 30 min to let them calm down. Subsequently, 3–5 min long videos of active exploration were recorded. The captured video sequences were preselected and then reviewed in an observer blinded setting. Selection criteria were stable position of the rat's head and active whisking.

### Muscle Harvesting

**Figure 4:**
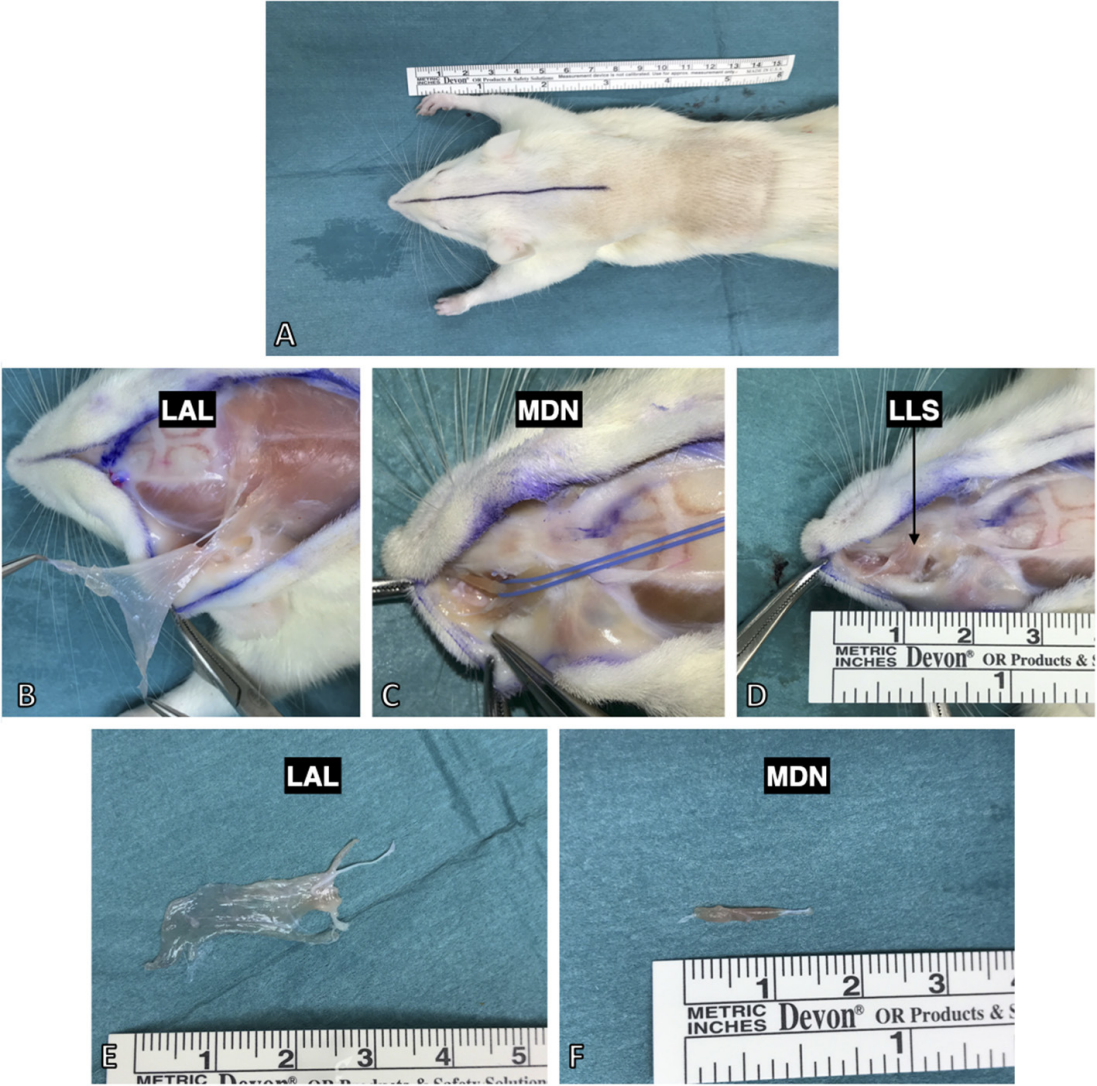
Harvesting procedure for the facial muscles after full body PFA perfusion. **(A)** All three were approached by a median incision from the nasal tubercles to the 6th cervical vertebra. **(B)** The levator auris longus muscle was isolated from its insertion at the auricle and lifted to its origin at the spines of the 1st−5th cervical vertebrae. **(C)** The dilator nasi muscle (indicated by the blue vessel loop) was accessed by lifting the levator labii superioris muscle. **(D)** The levator labii superioris muscle was isolated via blunt dissection from the maxilla and then followed to its intermingling in the whisker pad. **(E)** The harvested levator auris longus muscle with the innervating posterior auricular nerve still attached. **(E,F)** The isolated muscles demonstrated thin and flat anatomy making them suitable for various staining and imaging protocols (see Figure 6 for example).

Four weeks after surgery, the rats were deeply anesthetized with a terminal dose of ketamine and xylazine, followed by left ventricular perfusion with 400 ml of 0.9% NaCl and subsequently 400 ml of a 4% PFA solution. Three facial muscles (levator auris longus, levator labii superioris, and dilator nasi muscle) were exposed by a median incision from between the nasal tubercles to the caudal margin of the scapulae (Figure 4A). The levator auris longus (LAL) muscle was isolated first. Its cranial and caudal portions were each transected at their insertion points at the base of the auricle. Afterwards, the muscle was lifted and carefully separated from the trapezius muscle, located underneath it, toward its origin at the spines of the 1st−5th cervical vertebrae (Figure 4B). The levator labii superioris (LLS) muscle was isolated by blunt separation at its origin from the premaxilla and subsequent dissection ventralward to the mystacial pad (Figure 4D). Underneath the levator labii superioris muscle the delicate dilator nasi muscle was identified crossing parallel to the premaxilla toward the nostrils (Figure 4C). The dilator nasi muscle (MDN) was isolated by slinging a vessel loop around its tendinous part inserting at the nostril. Using the vessel loop, the muscle was lifted in an atraumatic manner allowing for further dissection toward its origin at the orbital edge of the maxilla. In five rats the muscles were also harvested on the contralateral, unoperated side (Figures 4E,F). The harvested facial muscles were stored in PBS containing 0.05% sodium azide to avoid bacteriological growth at +4°C for 24 h before staining.

### Whole-Mount Staining

The harvested muscles underwent whole-mount preparation and were multicolor immunolabeled. Prior to immunolabeling, the whole-mount preparations were frozen in ice cold (−80°C) 2-methylbutane and immediately thawed. Afterwards, they were kept in PBS containing 1% Triton X (PBS-T) for 24 h at room temperature. Both procedures were done to facilitate antibody penetration.

#### Triple Staining of Whole-Mount Preparations

Whole-mount preparations were tripe-labeled using antibodies against neurofilament (general marker for axons), α-bungarotoxin (neurotoxin that binds to the acetylcholine receptors of motor endplates) and phalloidin (mushroom poison that binds to actin filaments of muscle fibers). The tissue was incubated with 10% normal goat serum in PBS-T for 2 h at room temperature to block non-specific binding sites. This was followed by incubation with the primary antibody chicken anti-neurofilament (1:2000) for 48 h at room temperature. After extensive washing in PBS-T, the tissue was incubated with the secondary antibody Alexa flour 568 conjugated goat anti-chicken (1:500) along with Alexa flour 488 conjugated α-bungarotoxin and Alexa flour 647 conjugated phalloidin for 6 h at room temperature. Following washing in PBS-T, the tissue was embedded in glycerol-PBS.

#### Analysis of Labeled Whole-Mount Preparations

Labeled whole-mount preparations were analyzed using a confocal laser scanning microscope [CLSM (Olympus FV3000, Olympus Europa SE & Co. KG, Hamburg, Germany)]. A series of virtual CLSM sections of 1 μm thickness were cut through the structures of interest. Each section was photo-documented with a 1,024 × 1,024 pixel resolution and 3D projections were rendered using ImageJ (NIH, Bethesda, MA, USA). Triple-colored images were generated using lasers with excitation wavelengths 488, 568, and 633 nm.

## Results

### Surgery

All animals survived both the surgical procedure as well as the follow-up period without any adverse events. Mean operation time was 45 ± 12 min for the deefferentation procedure and 73 ± 17 min for the deafferentation procedure. The time needed for denervation of the individual afferent nerves was 15 ± 3 min for the mental, 11 ± 4 min for the mylohyoid, 24 ± 6 min for the infraorbital, and 21 ± 8 min for the supraorbital nerve. The anatomy of the afferent nerves in the rats was constant and without any variations. The qualitative functional assessment of the recorded video footage revealed successful denervation of the facial nerve with motor impairment of the whisker pad 4 weeks after surgery in all rats (Supplementary Video 1). Next to pathological immobility of the operated side, the rats displayed an atypical static orientation of the denervated vibrissae. In the deafferentation model, an ipsilateral anatomical deviation of the vibrissae was observed in rats following denervation of the infraorbital nerve (Supplementary Video 2).

### Axon Quantification

**Figure 5:**
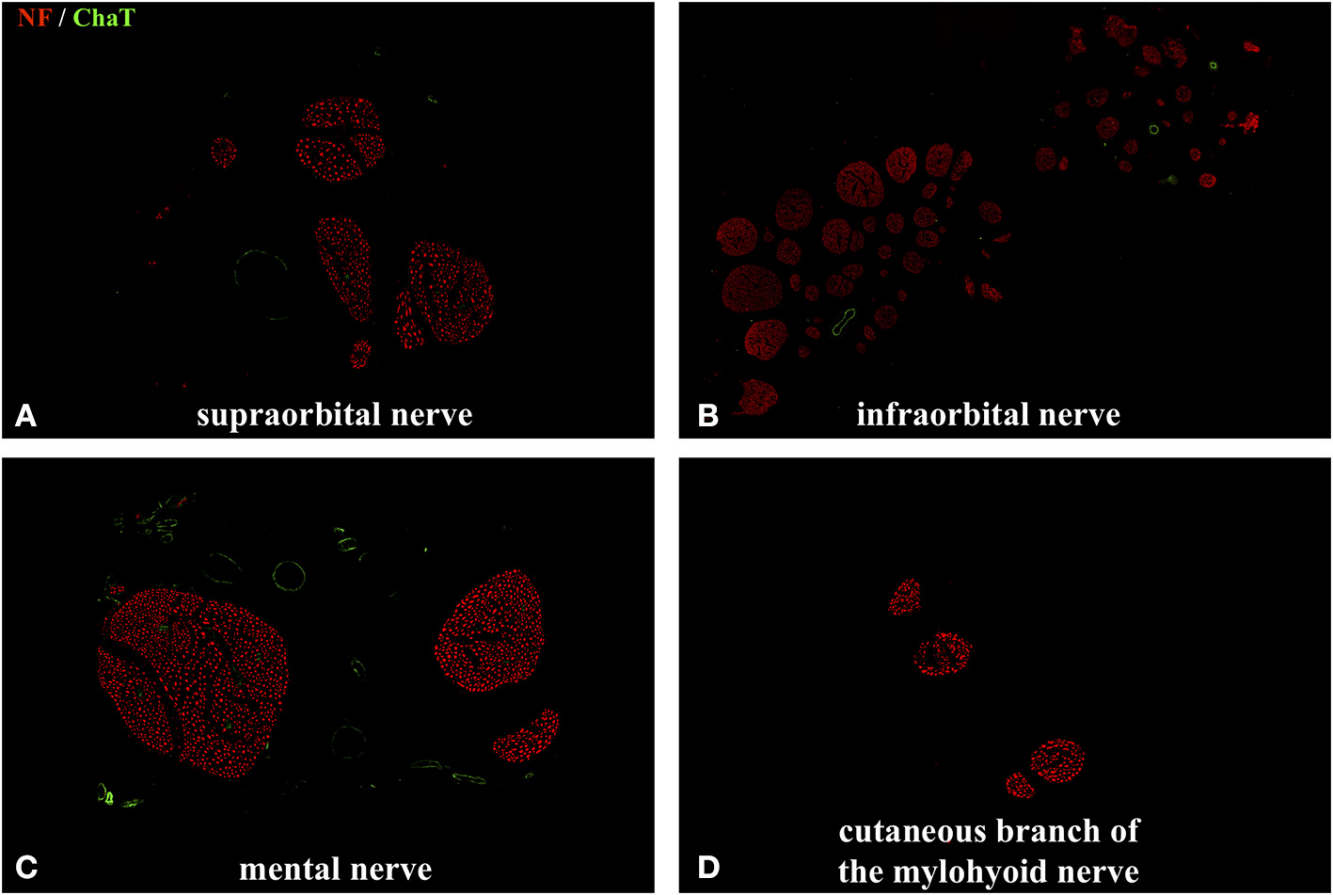
Immunofluorescent double staining of cross sections of the cutaneous branches of the trigeminal nerve using anti–choline acetyltransferase (ChAT) and anti-neurofilament (NF) antibodies. No colocalization of the ChAT with the NF signal was observed. **(B)** Axonal quantification showed the highest axon and fascicle number in the infraorbital nerve: 28,625 ± 2,519. **(A,C)** The supraorbital nerve contained 2,131 ± 413 axons and the mental nerve 3,062 ± 341. **(D)** As expected, the cutaneous branch of the mylohyoid nerve had the lowest axon count with 343 ± 78. No ChAT signal was detected in any of the afferent nerves.

We performed a comprehensive quantitative analysis of axons innervating the facial skin and whisker pad in the rat (Figure 5). Axons were quantified using antibodies against neurofilament, which is highly specific for axonal structures. This allows for both visualization and quantification of all axons within a nerve cross section (Gesslbauer et al., 2017). Axon count revealed 2,131 ± 413 in the supraorbital, 28,625 ± 2,519 in the infraorbital, 3,062 ± 341 in the mental and 343 ± 78 in the cutaneous branch of the mylohyoid nerve (Table 1). Fascicle number quantification revealed 5 ± 2 in the supraorbital, 61 ± 7 in the infraorbital, 3 ± 1 in the mental and 4 ± 1 in the cutaneous branch of the mylohyoid nerve (Table 1). None of the axons in the cutaneous branches of the trigeminal nerve displayed a choline acetyltransferase signal, thus confirming their pure afferent nature.

**Table 1:** Total axon and fascicle count of cross-sections of the individual cutaneous branches of the trigeminal nerve.

**Nerve (*n* = 5)**	**Total axons**	**Fascicles**
Supraorbital nerve	2,131 ± 413	5 ± 2
Infraorbital nerve	28,625 ± 2,519	61 ± 7
Mental nerve	3,062 ± 341	3 ± 1
Cutaneous branch of the mylohyoid nerve	343 ± 78	4 ± 1

*Data is presented as mean ± standard deviation*.

### Muscle Harvesting and Whole-Mount Staining

We demonstrated an efficient harvesting procedure for three facial muscles which may be utilized for multiple investigative purposes. In our experience, the dissection procedure's learning curve is steep, but allows for clear isolation and harvesting of the levator auris longus, levator labii superioris, and dilator nasi muscles by one surgeon and one assistant (Figure 4).

**Figure 6:**
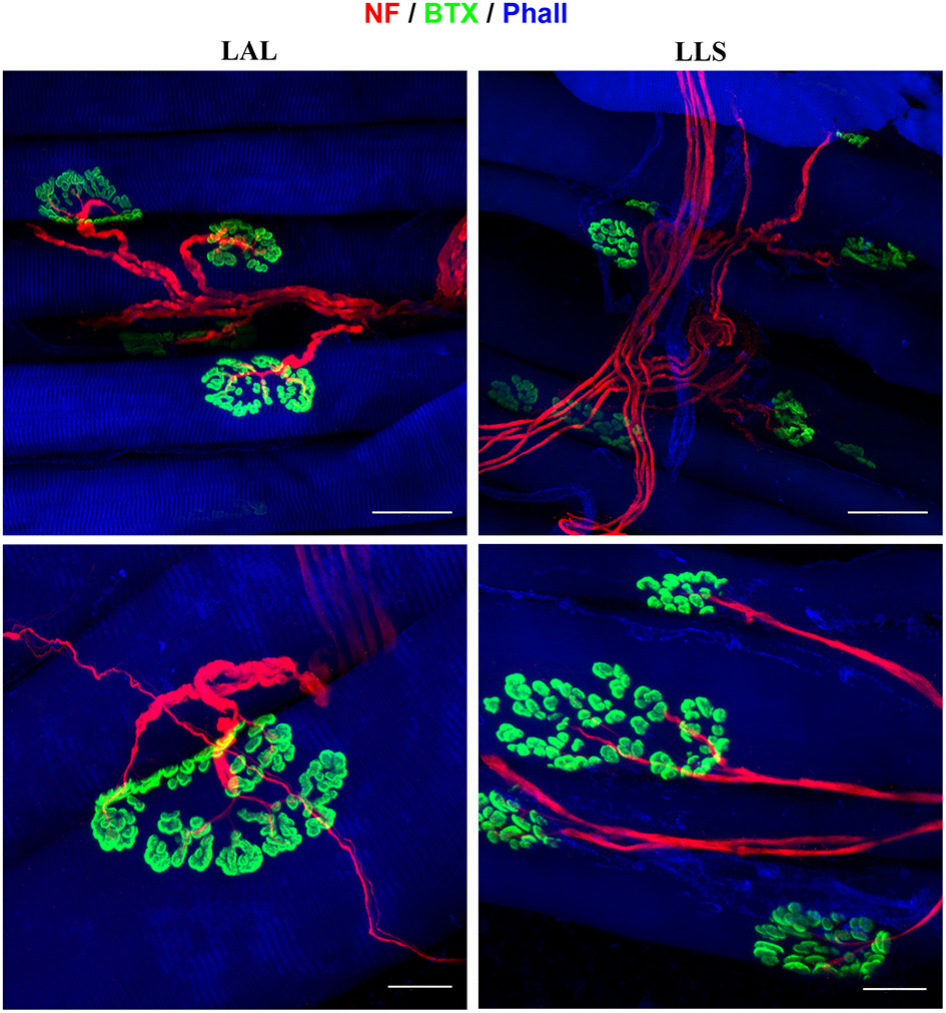
Immunofluorescent whole-mount staining of facial muscles using anti-neurofilament (NF), alpha-bungarotoxin (BTX), and anti-phalloidin (Phall) antibodies. **Left**: The levator auris longus (LAL) muscle was not subjected to any denervation procedure and displays parallel muscle fibers as well as a clear innervation pattern of the neuromuscular junctions. **Right**: The levator labii superioris (LLS) muscle was stained 4 weeks after denervation of the cutaneous branches of the trigeminal nerve. No alterations of the neuromuscular junctions' innervation were identified compared to unoperated control samples.

Due to the facial muscles' thin morphology, whole-mount immunofluorescent staining can also be applied to visualize both nerve branching patterns and neuromuscular junctions in the entire muscle. We demonstrated this approach for visualization of the innervation of neuromuscular junctions in three facial muscles. The use of antibodies against phalloidin revealed the parallel distribution of muscle fibers along the whole length of the dilator nasi muscle. All harvested muscles from the unoperated side stained with antibodies against alpha-bungarotoxin revealed clear central distribution of neuromuscular junctions (Figure 6). No micromorphological alterations of neuromuscular junctions were observed in any of the facial muscles 4 or 12 weeks after denervation of the trigeminal branches. Furthermore, neither polyinnervation nor denervation of neuromuscular junctions were identified after trigeminal denervation. Various combinations of antibodies can be applied using whole-mount staining allowing for the investigation of neurobiological processes within the facial muscles of the rat.

## Discussion

In this study, we established novel surgical models for selective denervation of facial muscles or skin in the rat. We demonstrated feasible and reproducible surgical procedures, which may be applied to explore a wide range of research questions. Specifically, it may aid to investigate the interplay between the afferent and efferent nerval innervation of the face, which is not entirely understood yet. The facial anatomy of rats presents very advantageous model for the investigation of the trigemino-facial system, as the whisker pad muscles are innervated afferent as well as efferent (Bendella et al., 2011). For subsequent analyses of the models, we demonstrated a whole-mount immunofluorescent staining protocol for these facial muscles to investigate their neuromuscular innervation and regeneration. In line, we provide a first quantification analysis of afferent axons innervating the rat's whisker pad and facial skin using a reproducible immunofluorescent staining protocol.

In regard to reproducibility of surgical models, the recorded video footage showed successful persistent denervation with motor impairment of the whisker pad 4 weeks after facial nerve denervation (Supplementary Video 1). In our experience, only once did an intraoperative complication occur in one rat: major bleeding from the superficial temporal artery during dissection of the facial nerve. With sufficient microsurgical skills, the models are highly reproducible following a short learning period, however, certain aspects require proper attention. In the deafferentation model, one must carefully dissect the infraorbital nerve superficial to the masseteric muscle to prevent damaging the distal pes of the facial nerve as it is located 0.5 mm rostral to the masseteric muscle (Henstrom et al., 2015). To identify the supraorbital nerve, various incisions are available. In our experience a median incision along the skull should be preferred over a supraciliary incision. Although, the latter approach is common in clinical routine for approaching the supraorbital foramen, it bears the risk in small rodents of accidentally damaging the distal ramification of the supraorbital nerve. In the deefferentation model, the preauricular approach to the facial nerve provides several advantages compared to other surgical approaches. The dissection is generally safe and fast with a clear view of all distal branches of the facial nerve and the postoperative course is typically uneventful. However, this approach does have a steep initial learning curve (Ali et al., 2020) and requires careful dissection to prevent bleeding from the superficial temporal artery or parotid gland fistula. In our experience, this can be prevented by blunt dissection of the glands and by using a vessel loop to manipulate the facial nerve. Despite this surgical approach being proposed in various facial nerve injury models for the investigation of nerve regeneration, no detailed description including figures of this surgical procedure has been provided so far (Guntinas-lichius and Hundeshagen, 2007; Magill et al., 2010; Placheta et al., 2015a; Liu et al., 2018).

The face is unique in its physiology, as its muscles and almost the entire facial skin are innervated exclusively by distal branches of a single cranial nerves: the facial and trigeminal nerves, respectively (Hwang et al., 2005; Ryan and Fee, 2009; Liu et al., 2019). Due to the separate innervation of the face's skin and muscles, understanding the interplay between the afferent and efferent systems is of great clinical interest for the pathophysiology and treatment of various diseases. In our current understanding, the face's innervation pattern is highly sophisticated due to distal connections between the facial and trigeminal nerves (Diamond et al., 2011; Hwang et al., 2015). However, despite the well-studied topography and anatomical variations of trigemino-facial interconnections, their functional significance is still not fully understood (Baumel, 1974). There is previous work, describing facial denervation (Pavlov et al., 2008; Sinis et al., 2009; Skouras et al., 2009), but to our knowledge, we demonstrated for the first time a comprehensive surgical approach for the denervation of all distal cutaneous branches of the trigeminal nerve in a rat model. It allows for the investigation of the facial skin and whisker pad completely deprived of afferent input. The whisker pad represents a sensory apparatus of huge interest in the fields of behavioral studies, cognitive plasticity and biomechanics (Guntinas-Lichius et al., 2002; Tomov et al., 2002; Seitz et al., 2011; Mameli et al., 2017; Yang et al., 2019). Furthermore, our denervation model prevents any aberrant reinnervation of the whisker pad from other afferent branches of the trigeminal nerve. Next to the terminal branches of the trigeminal nerve, we considered the afferent input from the cutaneous branch of the mylohyoid nerve to the facial skin as well. Interestingly, despite its surgical accessibility, this afferent nerve is rarely used in experimental animal models (Takahashi and Kimura, 1990; Yasuda et al., 1995). However, any damage inflicted to this nerve when performing procedures such as genioplasty or submandibular salivary gland removal in clinical practice may cause paresthesia of the submental skin area (Marinho and Tennant, 1997; Guyot et al., 2002; Hwang et al., 2005; Varol et al., 2009). Overall, this unique system of sensory and motor nerves innervating facial structures presents a useful tool to investigate interactions between afferent and efferent components during regeneration (Phillips et al., 2003; Bendella et al., 2011). Moreover, the effect on cortical plasticity after selective deefferentation or deafferentation is still poorly understood (Klingner et al., 2014). Due to the mixed quality (afferent/efferent) of most peripheral nerves, research into disruption of sensory-motor cortical connectivity after peripheral nerve injury or limb amputation cannot be interpreted easily in standard models (Qiu et al., 2014). Hence, our proposed surgical models allow distinctive investigation of motor-sensory mismatch on the cortical level.

To provide the necessary anatomical foundation of such investigations, we quantified the number of axons within the different cutaneous branches of the trigeminal nerve. Our data showed an unequal distribution between these branches using a highly specific immunofluorescent staining procedure. Previous studies using conventional histomorphometric analysis demonstrated similar quantitative data for the infraorbital (33,002 axons) (Jacquin et al., 1984), but not for the mental nerve (6,250 axons) (Johansson et al., 1992). In line, we performed a detailed surgical dissection of the cutaneous branch of the mylohyoid nerve and quantified its total axon composition, which has not been conducted in the rat. Overall, this data may be helpful in various experimental models for surgical nerve reconstruction models: the supraorbital and infraorbital nerves may be used for corneal neurotization (Catapano et al., 2018; Rosenblatt et al., 2020) as well as for sensory protection of cross-face nerve grafts for facial reanimation (Placheta et al., 2015b; Catapano et al., 2016) and the mylohyoid nerve has been proposed as a donor nerve for facial nerve reconstruction (Tubbs et al., 2007). A better understanding of the neurophysiological processes occurring as a result of the reconstructive procedures may optimize surgical outcomes in clinical practice.

Similar to the entire facial physiology, also the facial muscles have distinct innervation properties not seen in other skeletal muscles. These specific muscle properties are both highly relevant to investigate and at the same time allow for specific analyses not feasible in other skeletal muscles. Due to their specific anatomy, they are suitable for application of various research methods in neuromuscular research (Sinis et al., 2009). In this study we demonstrated a feasible harvesting procedure for three facial muscles in rat.

Compared to skeletal muscles of the extremity in the rat, facial muscles are morphologically flat and thin muscles making them optimal specimens for various staining and imaging procedures. In addition, the dilator nasi muscle is an easily identifiable and accessible muscle with a long rostral tendon, which may be utilized for various electrophysiologic studies (Weinberg et al., 2016). Our proposed approach for dissecting this muscle spares the levator labii superioris muscle's integrity allowing for simultaneous stimulation of both muscles. The levator auris longus muscle is thin and large, which allows for the use of epimysial multi-channel EMG electrodes to detect motor neuron activity in a highly specific manner (Muceli et al., 2019). Next to the aforementioned eligibility of facial muscles for various staining and electrophysiological methods, the atraumatic manipulation of the levator labii superioris muscle allows for the investigation of the whisker pad as it is one of the major muscles controlling the movements of this complex apparatus. There are several different approaches for investigating the innervation pattern of a muscle after nerve regeneration. One is the Sihler staining protocol, which enables mapping of the complete nerve supply within a muscle and demonstrates more consistent findings in thin skeletal muscles (Mu and Sanders, 2010). It furthermore enables the examination of the nature of trigemino-facial interaction at the muscular level after denervation procedures (Yang et al., 2013). Diverse immunofluorescent staining protocols may be used for investigating the neuromuscular regeneration at the cellular level (Ojeda et al., 2020). The implementation of imaging procedures of the facial muscles allows scanning of complete muscle thickness for the comprehensive visualization.

Due to discrepancies in the anatomical nomenclature of muscles controlling the whisker pad in the rat, we need to clarify the terminology we used in this study. We acknowledge recently proposed changes to the anatomical terminology (deflector nasi instead of dilator nasi and nasolabialis instead of levator labii superioris) in physiological, functional and immunohistochemical studies in the rat (Haidarliu et al., 2010; Deschênes et al., 2015). However, to avoid any confusion for readers we resorted to the anatomical nomenclature, which is commonly used in facial nerve and whisker pad research (Heaton et al., 2014; Weinberg et al., 2016).

In conclusion, we demonstrate a reproducible surgical protocol to denervate facial structures of afferent or efferent input in the rat model. This approach allows for efficient identification of both afferent branches of the trigeminal as well as distal branches of the facial nerve, which may be used for a wide variety of research questions. For the first time, we quantified the axonal composition of all cutaneous branches of the trigeminal nerve in the rat using an immunofluorescent staining method. We performed a universal harvesting method and established a highly specific whole-mount staining method for facial muscles in the rat, which allows for the investigation of neurobiological processes at a micromorphological level. This surgical model as well as the methods demonstrated in this study aid to further investigate the facial dermato-muscular matrix and thus understand their physiology and improve reconstructive approaches in the treatment of facial pathologies.

## Data Availability Statement

The raw data supporting the conclusions of this article will be made available by the authors, without undue reservation.

## Ethics Statement

The animal study was reviewed and approved by Medical University of Vienna and the Austrian Ministry for Research and Science.

## Author Contributions

VT, UM, OA, RB, and KB: conception and design. VT, DD, UM, CF, ML, GL, OP, OA, and KB: surgical procedure. VT, UM, CF, ML, OP, RB, OA, and KB: methodology. VT, UM, and RB: imaging. VT, DD, UM, RB, OA, and KB: data curation. KB, RB, HK, and OA: supervision. VT, DD, CF, HK, OA, and KB: drafting of the article. VT, DD, and RB: visualization. All authors critical revision and final approval of the version to be published.

## Conflict of Interest

The authors declare that the research was conducted in the absence of any commercial or financial relationships that could be construed as a potential conflict of interest.

## Supplementary Material

Supplementary Video 1 Video recording of whisker pad movement in a rat 4 weeks after facial nerve denervation.

Supplementary Video 2 Video recording of whisker pad movement in a rat 4 weeks after denervation of the cutaneous branches of the trigeminal nerve.
